# Visual Acuity Improvement in Continuous *vs* Divided Occlusion in Anisometropic Amblyopia

**DOI:** 10.2174/1874364101812010001

**Published:** 2018-02-14

**Authors:** Irawati Irfani, Feri Feriyanto, Primawita Oktarima, Arief Kartasasmita

**Affiliations:** Department of Ophthalmology, Universitas Padjadjaran/Cicendo Eye Hospital, Bandung, West Java, Indonesia

**Keywords:** Part-time occlusion, Full time occlusion, Amblyopia anisometropia, Visual acuity, Divided occlusion, Continuous occlusion

## Abstract

**Purpose:**

To compare visual acuity improvement between continuous and split part-time occlusion for the treatment of moderate and severe anisometropic amblyopia.

**Methods:**

Randomised clinical trials in 6 – 13 y.o children with moderate and severe anisometropic amblyopia. Each patient was consecutively selected with continuous or split part-time occlusion. Best corrected visual acuity’s improvement was followed up to six weeks and statistical data were analyzed using chi square and unpaired t-test.

**Results:**

Best corrected visual acuity’s improvement was comparable between continuous and split part-time occlusion (0.20±0.27 *vs* 0.21±0.25; p = 0.79).

**Conclusion:**

Split part-time occlusion may be considered as an alternative treatment for moderate and severe anisometropic amblyopia treatment.

## 1 INTRODUCTION

Occlusion therapy is still the treatment of choice for anisometropic refractive amblyopia [1, 2]. Amblyopia may cause permanent universal vision loss in children. The prevalence of amblyopia in school-aged children in the world is 2% [1, 3-5]. Compliance is an important aspect in undergoing occlusion therapy. Several studies were conducted to increase treatment compliance for occlusion therapy, by dividing the occlusion half time continuously or divided [6-8]. This study aimed to compare the increase of visual acuity on moderate and severe anisometropic amblyopia between continuous occlusion half time and divided occlusion half time in order to assess its effectiveness as an alternative therapy.

## 2 METHODS

The study was a controlled randomized clinical trial on patients with anisometropic refractive amblyopia aged 6 – 13 years from pediatric ophthalmology clinic in tertiary eye hospital, Bandung, Indonesia. The inclusion criteria were moderate and severe anisometropic amblyopia (with visual acuity in eye with amblyopia > 0.3 logMar, visual acuity difference between both eyes ≥ 0.2 unit logMar), had worn adjusted correction glasses for at least 4 weeks, had not received prior therapy for amblyopia. The exclusion criterion was patient being uncooperative during ophthalmological examination. The study was conducted upon approval from Ethics Committee of Medical Faculty Padjadjaran University.

The patients were divided into 2 groups using random blocks permutation, on the first group continuous half time occlusion was performed and on the second divided half time occlusion was performed. Occlusion was performed using patch treatment as shown in Fig. (**1**). Continuous occlusion was performed continuously for 4 hours a day and divided occlusion was performed for 2 hours twice a day with an interval of 2 hours. The observation was performed for 6 weeks, assessing the visual acuity after usage of correction glasses for 4 weeks, visual acuity was performed on 2^nd^, 4^th^, and 6^th^ week, while undergoing occlusion therapy. The visual acuity was evaluated using logMar chart from 4 meters distances. Compliance of treatment was observed by filling in the activity calendar accompanied with patients’ parents while undergoing occlusion therapy. Treatment compliance was divided into 3 categories: good (>85% occlusion time), moderate (70-85% occlusion time), and bad (<70% occlusion time).

Statistical analyses used in this study were Chi Square test and unpaired t-test. The significance of the study *p*< 0.05.

## 3 RESULTS

There were 30 subjects in the continuous occlusion group and 31 subjects in the divided occlusion group. Characteristics of the subjects are shown in Table **1**.

Occlusion was performed on the subjects for 6 weeks with 2 different occlusion techniques: continuous half time occlusion and divided half time occlusion. The resulting visual acuity and mean of visual acuity increase is shown in Table **2**.

No significant differences were found in increase of visual acuity and visual acuity difference between continuous half time occlusion group and divided half time occlusion group. (0.35 ± 0.26 *vs* 0.38 ± 0.22 (p = 0.72) ; 0.20 ± 0.27 *vs* 0.21 ± 0.25 (p= 0.79). The end result of visual acuity and mean of increase of visual acuity on moderate and severe amblyopia groups are shown in Table **3**.

Average of visual acuity in moderate amblyopia group after 6 weeks of occlusion treatment in group A was 0.22±0.1 logMar and in group B was 0.20±0.1 logMar. In severe amblyopia, the mean visual acuity after 6 weeks of occlusion treatment in group A was 0.53±0.31 logMar and in group B was 0.47±0.21 logMar. There was no significant difference between the visual acuity of moderate and severe amblyopia in both groups (*p* = 0.64; p = 0.52). The average increase of visual acuity in moderate amblyopia after 6 weeks of occlusion therapy in group A was 0.25±0.05 logmar and in group B was 0.27±0.08 logMar. The average increase of visual acuity in severe amblyopia in group A was 0.30±0.14 logMar and in group B was 0.37±0.15 logMar. There was no significant difference in increase of visual acuity between moderate and severe amblyopia in both groups. (p = 0.42; p = 0.19).

## 4 DISCUSSION

Kane *et al*., had compared treatment compliance of continuous occlusion and divided occlusion therapy and had shown the identical increase of visual acuity in both groups (p = 0.82). [9] This study has shown that increase of visual acuity was not significantly different between both treatments (0.35±0.26 logMar *vs* 0.38±0.22 logMar; p = 0.72). This is different with the study conducted by Bhoompally *et al*, whose result was the visual acuity in the group that had received continuous occlusion therapy was better than the group that had received divided occlusion therapy (0.51±0.26 logMar *vs* 0.59±0.04 logMar; p = 0.27). [6] Different results were also acquired compared to the same study, in the group that had received continuous occlusion therapy had a higher increase of visual acuity compared to the group that had received divided occlusion therapy. (0.47±0.04 logMar *vs* 0.37±0.05 logMar; p = 0.15) [6] This study had shown that the mean of visual acuity increase on the continuous occlusion group was not significantly different compared to the divided occlusion group. (0.21±0.25 logMar *vs* 0.20±0.27 logMar; p = 0.79).

This study had shown that, in moderate amblyopia, the mean of end visual acuity and the increase of visual acuity was higher in the divided occlusion group compared to the continuous occlusion group after 6 weeks of treatment. Meanwhile, in severe amblyopia, there was a difference, although statistically insignificant, in mean of end visual acuity and the increase of visual acuity between both groups.

The end visual acuity and mean increase of visual acuity in severe amblyopia was higher in divided occlusion group compared to continuous occlusion group. This may be caused due to higher interocular visual acuity difference in the divided occlusion group compared to the continuous occlusion group (the visual acuity in amblyopic eye is worse compared to continuous occlusion group), making the occlusion therapy effects on visual acuity becoming more noticeable in this group.

Several alternatives to improve comfort during occlusion therapy is to minimalize occlusion time by dividing the occlusion time and finding an alternative method for occlusion. One of them is by dividing occlusion time into several parts to increase treatment compliance. One of the most critical aspect in amblyopia treatment successistreatmentcompliance.

According to a study conducted by Al-Zuhaibi *et al*. suggested that with bad treatment compliance affect success chance in treating amblyopia, with increase of visual acuity was strongly associated with treatment compliance in occlusion therapy (p = 0.08) [10]

A study conducted by ATS 2B had found that the end visual acuity and increase of visual acuity in patients with moderate amblyopia in occlusion half time for 2 hours was as good as 6 hours of occlusion half time [7]. Identical results were acquired in severe amblyopia, according to a study conducted by ATS 2A, that had found the end visual acuity and increase of visual acuity in patients with severe amblyopia were better in half time occlusion for 6 hours compared to full occlusion.^7^ This study had indicated that shorter duration of occlusion increases therapy compliance. Singh *et al*, found that half time occlusion on mild amblyopia for 2, 4, and 6 hours were identically effective with full occlusion, while in severe amblyopia it has been shown that full occlusion and 6 hours occlusion were more effective compared to 2 hours occlusion [11].

Evaluated from compliance aspect, Jessica Kane *et al*. found the compliance in continuous occlusion group to be better compared to divided occlusion group (p = 0.023) [9]. According to a study conducted by Bhoompally *et al*. however, the compliance in divided occlusion (82%) was better compared to continuous occlusion (75%) [6]. The result differed with this study, where the compliance in continuous occlusion group (92%) was better compared to divided occlusion group (88%) although the difference was not statistically significant (p = 0.24). The difference of compliance levels may be caused by the duration of observation on both of the studies. The study conducted by Bhoompally observed the patient for 6 months compared to 6 weeks (which was performed in this study). Occlusion therapy for a long period of time may cause discomfort due to usage of occluder, thus reducing the treatment compliance. Additionally, usage of half time occlusion therapy may fit better to the patients’ schedule. In this study, the compliance was found to be good and moderate with an increase of visual acuity occurred in subjects with moderate and severe amblyopia.

The limitations of the study were the small sample size and relatively short duration of observation. The difference in adaptation period of corrective glasses (between 4 -16 weeks) may be a confounding factor in the increase of visual acuity in occlusion therapy.

## CONCLUSION

In this study, the half time occlusion therapy resulted in an increase of visual acuity identical with full time occlusion therapy in anisometropic moderate and severe amblyopia. There were no difference in compliance between the group with continuous half time occlusion and the group with divided half time occlusion. Half time occlusion therapy may be used as an alternative for anisometropic moderate and severe amblyopia therapy.

## Figures and Tables

**Fig. (1):**
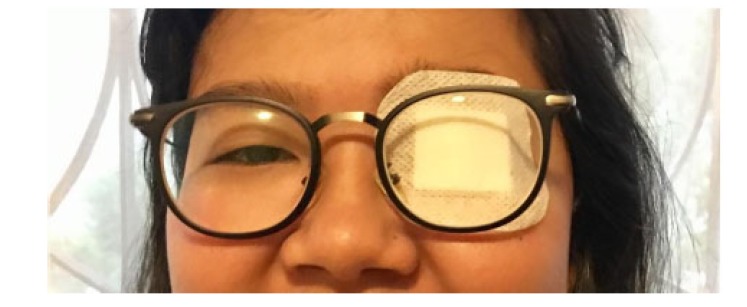
Patch treatment.

**Table 1:** Characteristics of study subjects.

**Characteristics**	**Continuous half time** **n=30**	**Divided half time** **n=31**	**P-value**
**Gender** • Men • Women	15 (50%) 15 (50%)	15 (48%) 16 (52%)	0.90
**Age (years)** • Mean ± SD	9.9 ± 2.5	9.6 ± 2.4	0.64
**Mother’s education** • Primary school • Middle school • Secondary school • Graduate / postgraduate	5 (17%) 7 (23%) 13 (43%) 5 (17%)	6 (19%) 5 (16%) 15 (49%) 5 (16%)	0.91
**Refraction adaptation (weeks)**	4.9 ± 0,5	4.6 ± 0.5	0.66
**Amblyopia severity** • Moderate • Severe	17 (61%) 13 (39%)	11 (39%) 20 (61%)	0.10
**Initial visual acuity (logMar)** • Dominant eye (Mean ± SD) • Amblyopic eye (Mean ± SD) • Interocular visual acuity difference (Mean ± SD)	0.24 ± 0.11 0.63 ± 0.25 0.40 ± 0.27	0.22±0.14 0.71 ± 0.26 0.48 ± 0.27	0.68 0.26 0.20
Compliance	92% ± 18%	88% ± 16%	0.45

**Table 2:** Initial visual acuity and increase of visual acuity of amblyopic eye in both groups after 6 weeks of treatment.

**Visual acuity variable (logMar)**	**Continuous half time** **n=30**	**Divided half time** **n=31**	**P-value**
Visual acuity (Mean ± SD)	0.35 ± 0.26	0.38 ± 0.22	0.72
Increase of visual acuity (Mean ± SD)	0.20 ± 0.27	0.21 ± 0.25	0.79

**Table 3:** Visual acuity and increase of visual acuity in amblyopic eye after 6 weeks Of treatment according to amblyopia severity.

**Variables** **Amblyopia severity**	**Groups**		**P- values**
	**A** **(n=30)**	**B** **(n=31)**	
**Moderate (0.3-0.6 logMar) (n=28)**	0.22 ± 0.1	0.20 ± 0.1	0.64
**Increase of visual acuity (Mean±SD)**	0.25 ± 0.05	0.27 ± 0.08	0.42
**Severe (> 0.6 logMar) (n=33)**	0.53 ± 0.31	0.47 ± 0.21	0.52
**Increase of visual acuity (Mean±SD)**	0.30 ± 0.14	0.37 ± 0.15	0.19

Notes: A: Continuous half time occlusion B: Divided half time occlusion

**Table 4:** LogMar conversion.

**Foot**	**Metre**	**Decimal**	**LogMAR**
**20/200**	6/60	0.10	1.00
**20/160**	6/48	0.125	0.90
**20/125**	6/38	0.16	0.80
**200/100**	6/30	0.20	0.70
**20/80**	6/24	0.25	0.60
**20/63**	6/19	0.32	0.50
**20/50**	6/15	0.40	0.40
**20/40**	6/12	0.50	0.30
**20/32**	6/9.5	0.63	0.20
**20/25**	6/7.5	0.80	0.10
**20/20**	6/6	1.00	0.00
**20/16**	6/4.8	1.25	-0.10
**20/12.5**	6/3.8	1.60	-0.20
**20/10**	6/3	2.00	-0.30
